# Visible-Light-Driven Photocatalytic Inactivation of Bacteria, Bacteriophages, and Their Mixtures Using ZnO-Coated HDPE Beads as Floating Photocatalyst

**DOI:** 10.3390/ma15041318

**Published:** 2022-02-10

**Authors:** Marius Urbonavicius, Sarunas Varnagiris, Simona Tuckute, Sandra Sakalauskaite, Emilija Demikyte, Martynas Lelis

**Affiliations:** 1 Center for Hydrogen Energy Technologies, Lithuanian Energy Institute, 3 Breslaujos, 44403 Kaunas, Lithuania; sarunas.varnagiris@lei.lt (S.V.); simona.tuckute@lei.lt (S.T.); martynas.lelis@lei.lt (M.L.); 2 Department of Biochemistry, Faculty of Natural Sciences, Vytautas Magnus University, 44404 Kaunas, Lithuania; sandra.sakalauskaite@vdu.lt (S.S.); emilija.demikyte@vdu.lt (E.D.)

**Keywords:** floating photocatalyst, visible light, ZnO films, reactive magnetron sputtering, Ni underlayer, *S. Typhimurium*, *M. Luteus*, bacteriophages

## Abstract

Semiconductor materials used as photocatalysts are considered among the most effective ways to treat biologically polluted water. Certainly, efficiency depends on the selection of photocatalyst and its substrate, as well as the possibility of its application in a broader spectrum of light. In this study, a reactive magnetron sputtering technique was applied for the immobilisation of ZnO photocatalyst on the surface of HDPE beads, which were selected as the buoyant substrates for enhanced photocatalytic performance and easier recovery from the treated water. Moreover, the study compared the effect on the inactivation of the microorganism between ZnO-coated HDPE beads without Ni and with Ni underlayer. Crystal structure, surface morphology, and chemical bonds of as-deposited ZnO films were investigated by X-ray diffraction, scanning electron microscopy, and X-ray photoelectron spectroscopy, respectively. Visible-light-induced photocatalytic treatment was performed on the Gram-negative and Gram-positive bacteria and bacteriophages *PRD1*, *T4*, and their mixture. Higher bacteria inactivation efficiency was obtained using the ZnO photocatalyst with Ni underlayer for the treatment of *S. Typhimurium* and *M. Luteus* mixtures. As for infectivity of bacteriophages, *T4* alone and in the mixture with *PRD1* were more affected by the produced photocatalyst, compared with *PRD1*.

## 1. Introduction

Water is the most crucial natural resource. It is known that about 2.5% of total water is classified as fresh water, while only 0.002% is recognised as humanly accessible [1,2]. Various researchers agreed that the capability to access freshwater resources is and will be an even greater issue shortly in the future. Among various issues regarding the scarcity of fresh water, water pollution plays one of the most important roles. The wide variety of pollutants (e.g., bacteria, dyes, pharmaceuticals, pesticides, bacteriophages, etc.) produced due to human activities can negatively influence ecosystems as well as human health. Nowadays, conventional wastewater treatment processes (WWTPs) are generally used for wastewater treatment. Unfortunately, various reports are showing that such treatment often contains biological contaminants and traces of emerging chemical pollutants. Even more, some researchers considered WWTPs as gateways of biological contaminants into the aquatic environment or as hot spots for antibiotic resistance proliferation [3,4,5,6]. Such a possibility for these constituents to enter the environment is considered a severe public health issue including various diseases such as diarrhoea, vomiting, abdominal discomfort, gastroenteritis, etc. [7]. Therefore, various researchers focused on the investigation of novel and effective treatment methods, which could detoxify various biological contaminants.

In this regard, advanced oxidation processes (AOPs), such as heterogeneous photocatalysis with semiconductor materials, are recognised as an effective way in organic wastewater treatment. Among various semiconductor materials applied for photocatalytic biological contaminants inactivation, ZnO, which belongs to II-VI class of semiconductors, is depicted as one of the most promising materials. ZnO is known as a good photocatalyst for its optical and chemical properties, nontoxic nature, high exciton energy (60 meV) even at room temperature, and high oxidation capacity. Additionally, it is easy to grow or deposit a hexagonal ZnO wurtzite structure, which is a thermodynamically stable phase. Due to its environmentally friendly characteristic, ZnO is compatible with living organisms. Therefore, it has a broad range of daily applications by not leaving risks to human health and environmental impacts [8]. Meanwhile, during wastewater treatment or purification, ZnO can be adsorbed on the surface of biological contaminants and inhibit the growth of microorganisms [9]. However, the main drawbacks of ZnO are relatively high recombination of e^−^/h^+^ as a single system, wide bandgap nature (about 3.36 eV), and severity to be activated under daylight irradiation [10,11,12,13]. Only ~4% of the UV portion of solar energy can be utilised for ZnO photoexcitation in the photocatalytic process. Therefore, it is required to extend ZnO photoresponse into the visible-light region (~43% of solar spectrum) [14]. Additionally, due to the fast recombination of UV or visible light photogenerated electron–hole pairs, photocatalytic efficiency is suppressed [15]. Therefore, in order to separate electrons and holes, new energy levels in the bandgap need to be created. Various techniques are investigated to overpass these issues, including doping of metal/nonmetal elements, coupling with other semiconductors, deposition of noble metals, etc. [16,17,18,19,20,21]. For instance, Y. Jiang et al. investigated the possibility to immobilise zeolitic imidazole-based metal–organic framework into ZnO structure. They showed effective E. coli bacteria inactivation results under visible-light irradiation, while disinfection in water can be fully completed in 50 min [22]. Another group with Z. Mirzaeifard et al. analysed S-doped ZnO nanoparticles under visible-light irradiation for RhB degradation. They showed that such a combination could decompose 100% of RhB in 90 min. Moreover, the cycling experiment showed 92% decomposition after five consecutive reactions [23]. Meanwhile, some authors suggest that doping or other types of Ni and ZnO combination can be a suitable way to improve ZnO characteristics [24,25,26,27]. Moreover, our previous study revealed that Ni underlayer improves TiO_2_ photocatalytic efficiency, which was applied as floating photocatalyst for *Salmonella typhimurium* inactivation under visible-light irradiation [28].

Floating photocatalysts are an on-top option for wastewater treatment, where photocatalyst is immobilised on the floatable substrate. The main advantage of this technique against other photocatalyst methods is the possibility to employ the maximum light irradiation, which invokes intensive formation of reactive oxygen species (ROS) and enhances the photocatalytic performance [29,30]. Generally, floatable substrates have significantly larger sizes than powders. Therefore, the separation of such materials after wastewater treatment is a much easier process, compared with powdered photocatalysts. There are various articles showing improvement of photocatalytic performance using floating photocatalyst by decomposing various bacteria [31,32,33,34,35].

The analysis of one-type bacteria inactivation is a relatively simple and effective way to evaluate photocatalyst capability to decompose biological contaminants. Therefore, the majority of the research studies provide experiments regarding one type of contaminant (generally it is bacteria, such as *Escherichia coli* or *Salmonella enterica*). Still, the real condition wastewater might involve various types of diverse contaminants. Therefore, the investigation of biological contaminants mixture inactivation by photocatalytic materials is very important as well. Various articles have analysed diverse contaminants mixtures inactivation by photocatalysis. For instance, *Pseudomonas aeruginosa* and *Bacillus subtilis* [3], virus/bacteria system [36], organic micropollutants, human pathogen indicators, antibiotic-resistant bacteria and related genes [37], *Shigella species* and *Vibrio cholera* [38], *Mycobacterium kansasii*, and *Mycobacterium avium* [39], *Escherichia coli* and *Pseudomonas aeruginosa* [40], etc. mixtures were used for photocatalytic inactivation experiments. Unfortunately, there is a lack of scientific reports investigating the application of floating photocatalysts on the inactivation of biological contaminants mixtures. Moreover, the photocatalytic inactivation experiments of viruses or their mixtures are even more uncommon, because such experiments require a corresponding number of the specific host cells for their replication [41].

Therefore, in this study, we investigate photocatalytic inactivation of bacteria mixture and bacteriophages mixture initiated by a combination of visible-light and ZnO-based floating photocatalyst. Specifically, the bacteria mixture consists of Gram-negative (*Salmonella typhimurium*) and Gram-positive (*Micrococcus luteus*) bacteria. Meanwhile, the bacteriophages mixture involves *PRD1* and *T4*. The *T4* is a relatively stable bacteriophage, which reactivates under UV irradiation, while *PRD1* is a lipid envelope containing phage [42]. High-density polyethylene (HDPE) beads were selected as floatable substrates due to proper characteristics, which are listed elsewhere [43]. Part of HDPE beads was pre-covered by a thin nickel underlayer to test if it improves the photocatalytic activity in the case of ZnO. Our previous studies [28,44] showed that the metallic underlayer can positively affect photocatalytic performance under both UV–B and visible light irradiation. Additionally, some studies suggest that the recombination rate of electron–hole can be reduced and photocatalytic efficiency improved using metallic substrates and transition metals dopants [45,46,47]. Another study reported that photocatalytic performance (including charge separation and absorption of visible light) can be enhanced by coupling semiconductors (such as TiO_2_, ZnO, Cu_2_O, CdS, etc.) with metals [48]. In our case, the magnetron sputtering technique was applied for the ZnO photocatalyst immobilisation onto HDPE beads (with or without Ni underlayer), which, in our experience, is a suitable method for photocatalyst formation onto floatable substrates [49,50]. The obtained results revealed characteristics of both bacteria and bacteriophage mixtures inactivation using ZnO-based floating photocatalyst under visible-light irradiation.

## 2. Methodology

### 2.1. ZnO Film Deposition on the Floating Substrate

**Figure 1:**
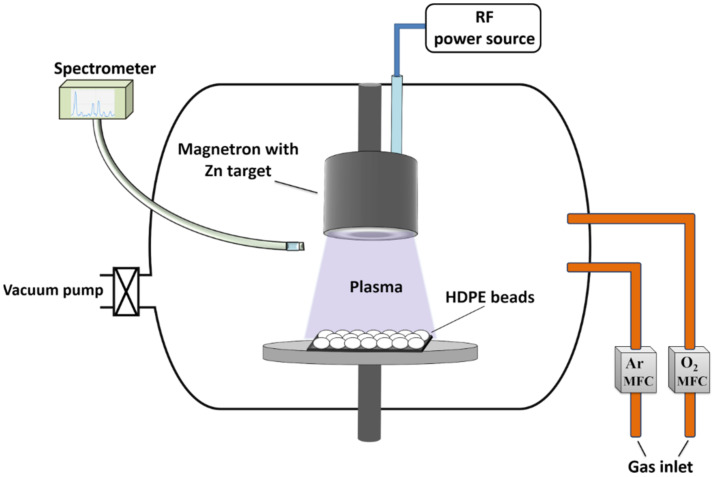
Experimental scheme for the deposition of ZnO films on the HDPE beads.

Deposition of ZnO films on the surface of HDPE beads (obtained from GoodFellow, Huntingdon, UK) with and without Ni underlayer was performed by reactive magnetron sputtering technique in a custom modified physical vapour deposition system (PVD-75, Kurt J. Lesker Company, Jefferson Hills, PA, USA) (Figure 1). Prior to the ZnO deposition, the HDPE beads were precovered with a thin layer of Ni (thickness of about 100 nm) using magnetron with Ni target (purity 99.99%, Kurt J. Lesker Company, Clairton, PA, USA) powered by a direct current power source. Our previous study disclosed that metallic underlayers can have positive impacts on the bactericidal effect of the photocatalyst under visible-light irradiation [28]. During the process, HDPE beads were placed under the unbalanced magnetron with a circular Zn target (99.99% purity and 76 mm diameter, Kurt J. Lesker Company, Clairton, PA, USA) which was powered by an RF power source working at 150 W for 1 h. After that, the HDPE beads were flipped over, and ZnO film was deposited on the other side of the beads under the same conditions. The distance between the HDPE beads and Zn target was 7 cm. The ZnO film was deposited at a fixed pressure of 1 × 10^−2^ mbar which was preselected experimentally as an optimal one according to our previous study [51]. Ar and O_2_ gas mass flow controllers (MFC) were connected to the Process Control and Plasma Emission Monitoring System (Flotron X, Nova Fabrica Ltd., Ignalina, Lithuania) which ensured a constant pressure and an accurate Ar and O_2_ gas flow ratio (4:1) according to the changes of Zn emission intensity. In order to measure the thickness of ZnO film, the film was deposited on the flat quartz substrate under similar reactive magnetron sputtering conditions. The approximate thickness of ZnO film measured by stylus profilometer (Ambios XP-200, Ambios Technology, Santa Cruz, CA, USA) was 4–5 µm.

### 2.2. Characterisation

The crystal structure of deposited ZnO film on the HDPE beads with and without Ni underlayer was characterised using an X-ray diffractometer (XRD, Bruker D8, Hamburg, Germany) operating with Cu Kα radiation (theta–theta configuration) in the 2Θ range of 25–70° and at the measurement step size of 0.01°. The crystallite size was calculated by Topas 6.0 software, using the Scherrer equation with Lorentzian deconvolution. The surface morphology of the samples was measured by scanning electron microscope secondary electron detector (SEM, Hitachi S3400N, Tokyo, Japan). The elemental mapping analysis was performed using an energy dispersive X-ray spectroscope (EDS, Bruker Quad 5040, Hamburg, Germany). X-ray photoelectron spectroscope (XPS, PHI 5000 Versaprobe, Boston, MA, USA) was used for surface elemental and chemical bond analysis. The XPS analysis was performed using the following parameters: monochromated 1486.6 eV Al radiation, 12.5 W beam power, 50 µm beam size, and 45° measurement angle. The optical bandgap of ZnO film was evaluated using an additional sample where ZnO film was deposited on a borosilicate glass substrate. Deposition conditions were kept the same as forming ZnO on HDPE beads. The Tauc plot calculation was applied for bandgap evaluation using obtained transmittance spectra, which were measured by an ultraviolet-visible (UV-VIS) spectrophotometer (Jasco V-650, Tokyo, Japan). The results revealed that obtained bandgap was about 3.08 eV, which is a significantly lower value, compared with the theoretical ZnO bandgap.

### 2.3. Bacteria Inactivation and Infectivity of Bacteriophages

#### 2.3.1. Bacteria Cultivation

For bacteria cultivation, 1–3 colony-forming units of *Salmonella enterica ser. Typhimurium SL1344* (Gram-negative, Institute of Food and Health, University College Dublin, Ireland) and *Micrococcus Luteus* (Gram-positive, Institute of Biosciences, Vilnius University, Vilnius, Lithuania) were inoculated in 10 mL of fresh LB medium and incubated at 37 °C for 18–20 h with shaking of 220 rpm. After incubation, the overnight culture was diluted in 50 mL of fresh LB medium, to obtain 0.2 of OD_600_, and incubated for about 2.5 h (for *S. Typhimurium*) and 3.5 h (for *M. Luteus*), to reach 0.8–1 of OD_600_. The obtained suspension was centrifuged at 3000× *g* for 10 min at 4 °C temperature (Heraeus™ Megafuge™ 16R, Thermo Scientific, Bremen, Germany) and resuspended in 400 µL of LB medium.

#### 2.3.2. Bacteria Inactivation Test

HDPE beads coated by ZnO were kept in the dark before the experiment. Tests of the viability of bacteria were carried out in 15 mL of phosphate-buffered saline (PBS) solution (Roth, Karlsruhe, Germany) in thermostated vessels with stirring (333 rpm) at 22 °C. Before adding mixtures of bacteria, 1 g of HDPE beads coated by ZnO was activated for 15 min under visible-light irradiation (Solis-3C, 5700 K, wavelength range of approximately 400–800 nm, Thorlabs, Dachau, Germany) at 65 mW/cm^2^. During the experiments, the concentration of *S. Typhimurium* and *M. Luteus* was 0.00001 and 0.00009 of OD_600_, respectively. After 2.5 h of treatment by visible-light irradiation (65 mW/cm^2^), 250 µL of the sample was taken from the vessel and diluted 5 times. Bacteria viability tests were performed by a spread-plate technique using 50 µL of the diluted sample which were spread by glass beads on LB–Agar in a Petri dish (Copacabana Method, see [52]). The viability of *S. Typhimurium* was assessed after 18–22 h of incubation at 37 °C without agitation and of *M. luteus*, after 36–44 h.

#### 2.3.3. Treatment of Bacteriophages

During the investigation, two types of bacteriophages were used—*PRD1* and *T4*—which hosts are *S. enterica* DS88 and *E. coli* DH5α, respectively. The experimental conditions were the same as performing the bacteria inactivation test. However, the mass of HDPE beads coated by ZnO was 2 g when mixtures of bacteriophages were used and 1 g when using separate suspensions. The concentration of bacteriophages was 1 × 10^4^ (pfu/mL). After 1 h of treatment by visible-light irradiation, 100 µL of bacteriophage suspension was taken from the vessel and diluted 10 times. Afterwards, 100 µL of obtained suspension was poured into an overnight culture of the host’s bacteria suspension. Finally, the suspension was spread on the LB–Agar Petri dish (see the plaque-forming method in [53]). The infectivity of bacteriophages was evaluated after 22 h of incubation at 37 °C.

## 3. Results and Discussion

### 3.1. Structural Analysis

**Figure 2:**
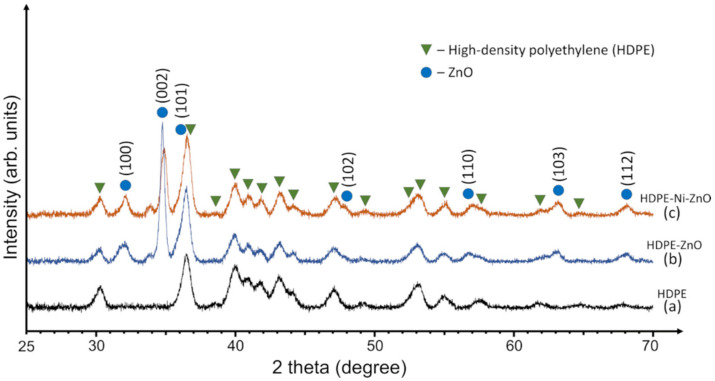
XRD patterns of (a) primary HDPE beads, (b) ZnO deposited on the HDPE beads without Ni underlayer, and (c) ZnO deposited on the HDPE beads with Ni underlayer.

Phase structure of primary HDPE beads and ZnO deposited on the HDPE beads without and with Ni underlayer was examined by X-ray diffraction technique (Figure 2). XRD pattern of primary HDPE beads is presented in Figure 2a. The size of beads used in this study was 2–4 mm. The XRD data were collected from the surface of HDPE beads which is exceptionally rough for XRD and, unlike the flat sample, causes the broadening in the peaks. However, all of the peaks matched the structure of the high-density polyethylene ((C_2_H_4_)_n_) which corresponds to the standard JCPDS card number 00-060-0984. Any other crystalline impurities were not detected.

The XRD results of ZnO deposited on the HDPE beads without and with Ni layer (Figure 2b,c, respectively) correlated well with the hexagonal wurtzite structure of ZnO (JCPDS card number 01-070-8070). The characteristic diffraction peaks of ZnO at 2Θ = 31.7°, 34.5°, 36.2°, 47.5°, 56.6°, 62.9°, and 68.0° correspond to (100), (002), (101), (102), (110), (103), and (112) crystal planes, respectively. This is totally consistent with the XRD analysis of ZnO reported in the literature [54,55,56]. It can be observed that the predominant crystal plane is (002), commonly found in the films deposited by sputtering technique due to the minimum surface energy of (002) which results in higher crystallites orientation rate along the c-axis [57,58,59]. The average crystallite sizes were 28 nm and 21 nm for the ZnO (002) deposited on HDPE beads without Ni and with Ni underlayer, respectively. Similar behaviour was found in the literature when various additional impurities tend to reduce the crystallite size slightly [23,60,61].

In the case of HDPE beads with Ni underlayer, XRD scanning did not detect any peaks attributed to nickel (Figure 2c). Presumably, Ni may have been deposited in an amorphous state, or the intensity of Ni peaks is too low to be visible in the spectrum. However, the XRD results confirm the successful deposition of crystalline ZnO onto a floatable substrate.

### 3.2. Morphology and Elemental Mapping Analysis

**Figure 3:**
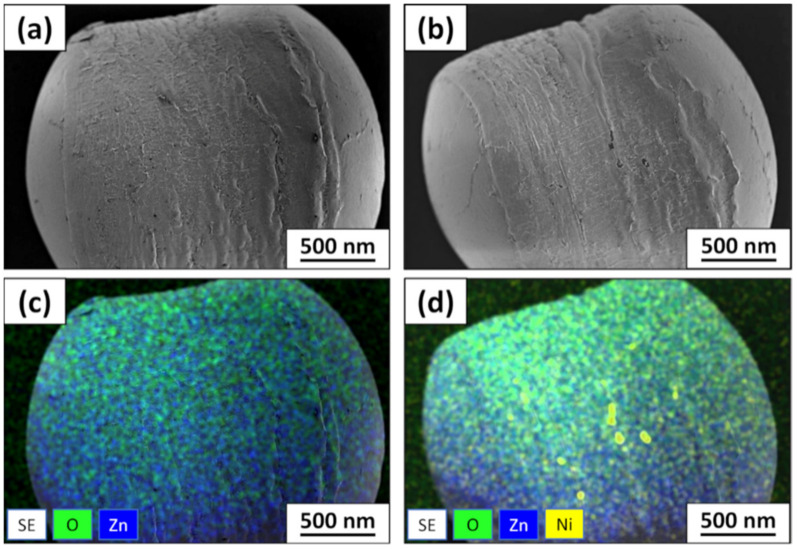
SEM views and EDS mapping of ZnO deposited on HDPE surface (**a**–**c**) without Ni underlayer and (**b**–**d**) with Ni underlayer, respectively.

The surface morphology of the floating ZnO–HDPE photocatalyst was investigated by SEM as shown in Figure 3. First of all, the images indicated that the surface of HDPE support was successfully deposited by ZnO film without significant defects, scarcities, or detachments of ZnO film. Still, deposited ZnO film results in roughening of the relatively smooth HDPE bead surface. These observations can be applied for both types of photocatalyst despite the existence of Ni underlayer (Figure 3a,b).

The elemental mapping confirmed the relatively uniform distribution of Zn and O elements across the HDPE beads surface in both cases. Figure 3d represents photocatalyst with Ni underlayer, while only a few Ni clusters can be observed. This is related to the ZnO film thickness, which is about 4–5 µm and technological limitations of EDS determine that Ni can be seen only in the areas with thinner ZnO film, its absence or cracks. In general, the surface morphology and elemental mapping analysis showed that Ni underlayer does not have a significant influence on ZnO film surface characteristics deposited on HDPE beads.

### 3.3. XPS Results

**Figure 4:**
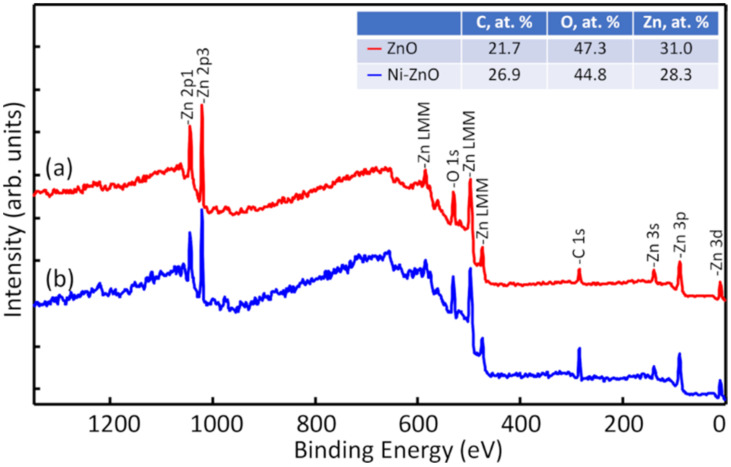
XPS survey spectra of ZnO deposited on the HDPE beads (a) without Ni and (b) with Ni underlayer.

Elemental surface analysis of ZnO film deposited on the HDPE beads without and with Ni underlayer is presented in Figure 4. In both cases, the detected elements were carbon (C), oxygen (O), and zinc (Zn), without any unwanted impurities. Quantification of these elements demonstrated similar results for both samples (inserted table in Figure 4). Such an amount of carbon is observed due to exposure of samples to the ambient air. After ZnO film deposition, samples were taken out from the vacuum chamber and handled in the air which caused the formation of a thin adventitious carbon/hydrocarbon (and/or hydroxyl species) layer [62]. The Ni underlayer is immobilised under a thick layer of ZnO. Therefore, the XPS did not detect any peaks of Ni since it is a surface-sensitive measurement technique.

**Figure 5:**
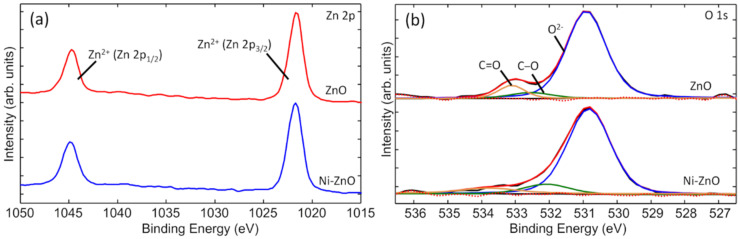
Comparison of (**a**) Zn 2p and (**b**) O 1s electron spectra for ZnO deposited on the HDPE beads without Ni (ZnO) and with Ni underlayer (Ni-ZnO).

The surface chemical bond analysis of the ZnO film deposited on the HDPE beads without and with Ni underlayer was performed by high-resolution XPS scans for Zn 2p and O 1s regions (Figure 5a,b, respectively). However, the Ni underlayer did not affect the chemical structure (oxidation state) of the surface because both samples showed nearly identical spectra. Zn 2p spectrum consisted of two main components of Zn 2p_3/2_ and Zn 2p_1/2_ positioned at 1021.8 and 1044.9 eV, respectively (Figure 5a). According to other studies, these binding energies and separation of 23.1 eV between two peaks represent the Zn^2+^ ions of ZnO lattice (Zn–O bonds) [63,64,65]. The O 1s electron spectrum was deconvoluted into 3 peaks (Figure 5b). The major one at about 530.8 eV was assigned to the O^2-^ oxidation state which also confirmed the formation of Zn–O bonds in the wurtzite structure. The shoulder peaks (at the higher energies) can be attributed to the oxygen atoms in C–O, C=O bonds, or even hydroxyl groups, due to surface contamination by species and moisture existing in the air [66].

### 3.4. Inactivation of the Mixture of M. Luteus and S. Typhimurium Bacteria

**Figure 6:**
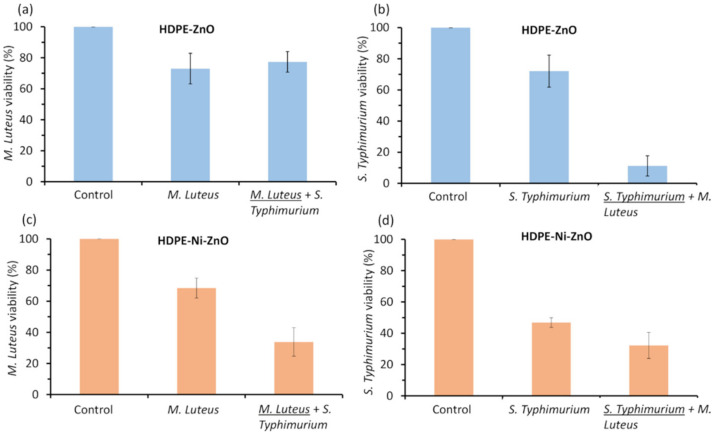
Photocatalytic inactivation of separate *M. Luteus* and *S. Typhimurium* bacteria and their mixture under visible-light irradiation for 2.5 h: (**a**) viability of *M. Luteus* alone and in the mixture with *S. Typhimurium* using ZnO film deposited on the HDPE beads without Ni underlayer; (**b**) viability of *S. Typhimurium* alone and in the mixture with *M. Luteus* using ZnO film deposited on the HDPE beads without Ni underlayer; (**c**) viability of *M. Luteus* alone and in the mixture with *S. Typhimurium* using ZnO film deposited on the HDPE beads with Ni underlayer; (**d**) viability of *S. Typhimurium* alone and in the mixture with *M. Luteus* using ZnO film deposited on the HDPE beads with Ni underlayer. The length of the error bars is the standard deviation of each measurement.

In this study, the bactericidal effect of the floatable photocatalyst was evaluated. The viability of *M. Luteus* and *S. Typhimurium* separately and a mixture of these two types of bacteria were tested under visible-light irradiation using ZnO film deposited on the HDPE beads without and with Ni underlayer (Figure 6).

Prior to the experiments, blank tests were performed without photocatalyst under visible light irradiation which showed that viability of bacteria at most decreased by approximately 20%. Meanwhile, the viability of bacteria with photocatalyst in the dark was not affected. Photocatalyst without Ni underlayer reduced the viability of *M. Luteus* approximately by about 25% when irradiated by visible light separately or in a mixture with *S. Typhimurium* (Figure 6a). The result was slightly better than the one obtained during the blank test without photocatalyst. On the other hand, after irradiation of *S. Typhimurium* bacteria the viability decreased by 89% when it was in the mixture with *M. Luteus* and only 28% while irradiating the suspension of *S. Typhimurium* individually (Figure 6b). Interestingly, the inactivation of separate bacteria showed similar values, but the results significantly differed when comparing bacteria mixtures.

The photocatalyst with Ni underlayer showed greater efficiency, compared with HDPE-coated by ZnO film without Ni underlayer. Presumably, Ni creates a synergistic effect on photocatalyst performance. Although the Ni metal underlayers were used in our case, it is known that transition metal dopants can introduce inter-bands in the photocatalyst structure and enhance the charge separation by suppressing the electron–hole recombination. [67,68]. Due to the thick ZnO film and limited measurement depth, the XPS and XRD analysis methods did not detect Ni underlayer. Nevertheless, it cannot be excluded that some Ni additives might be diffused in random parts of the ZnO structure. Presumably, the dopants can increase the concentration of oxygen vacancies in the structure which further enhances the bactericidal effect of the photocatalyst [69]. It was reported that transition metal-doped ZnO particles have a synergistic effect on photocatalytic and antibacterial performances [15,47,70]. On the other hand, the EDS mapping showed some spots of Ni in the areas where ZnO film is thinner or cracked. Accordingly, these Ni spots can partially contribute to the reduction in bacteria viability by causing oxidative stress due to the direct interaction with bacteria [71]. The viability of *M. Luteus* was reduced by 32% after irradiating a suspension individually and by 66% when in the mixture with *S. Typhimurium* (Figure 6c). Meanwhile, the *S. Typhimurium* bacteria were more sensitive than *M. Luteus* after individual treatment under visible light. The viability of *S. Typhimurium* decreased by 53% when bacteria were tested alone. However, obtained results were nearly the same as with *M. Luteus* after irradiation of bacteria in the mixture (the remaining viability was 32%) (Figure 6d).

Obtained results suggest that in the case of bacteria mixtures, the viability of bacteria depends on the bactericidal efficiency as well as on the interaction between different types of bacteria. For example, it is known that Gram-positive bacteria such as *Staphylococcus aureus* inhibit the growth of *Salmonella* [72]. Therefore, the increased bactericidal effect of photocatalyst against *S. Typhimurium* bacteria in the mixture with *M. Luteus* (Figure 6b) could be due to the negative interaction between two species of bacteria.

### 3.5. Inactivation of the Mixture of PRD1 and T4 Bacteriophages

**Figure 7:**
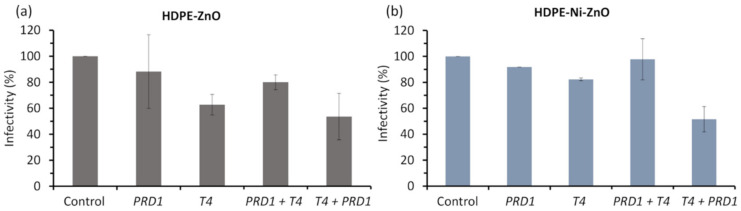
Infectivity of separate *PRD1* and *T4* bacteriophages and their mixture under visible-light irradiation for 1 h using ZnO film deposited on the HDPE beads (**a**) without Ni underlayer and (**b**) with Ni underlayer. The length of the error bars is the standard deviation of each measurement.

ZnO photocatalyst alone (in the dark) did not affect or lower the infectivity of bacteriophages. On the other hand, the blank test of visible light irradiation on bacteriophages without the photocatalyst showed that the infectivity decreased by about 2–7%. Results showed that bacteriophages seem more resistant to light than bacteria. ZnO-coated HDPE beads without Ni underlayer reduced the infectivity of *PRD1* phage by 12%, while in the case of mixture with *T4*, by 20% after irradiation under visible light (Figure 7a). Moreover, the infectivity of *T4* decreased by 38% and 47% after irradiation of *T4* alone and in the mixture with *PRD1*, respectively. To summarise, this investigation demonstrated that visible-light-activated ZnO photocatalyst (without Ni) had a more noticeable effect on the bacteriophage mixtures than on bacteriophages alone.

The obtained results were just slightly different while testing the ZnO-coated HDPE beads with Ni underlayer (Figure 7b). The infectivity of the *PRD1* phage decreased by about 10%, while the photocatalyst was inefficient against *PRD1* in the mixture with *T4* phage. The infectivity was reduced by 18% and almost 50% after treatment of *T4* alone and in the mixture with PRD1, respectively. Consequently, the *PRD1* bacteriophage is sufficiently resistant to ZnO photocatalyst deposited on the HDPE beads with Ni underlayer, and the sensitivity of T4 phage strongly increased when treated in mixture with the phage of *PRD1*. In both cases, the ZnO-based floating photocatalyst affects bacteriophages less than bacteria (Figure 6). It is known that bacteriophages (as viruses) are more resistant to light irradiation or stress effects than bacterial cells [73].

## 4. Conclusions

In the current study, HDPE beads with and without Ni underlayer were used as floatable substrates for the ZnO photocatalyst which was successfully deposited via reactive magnetron sputtering technique. In both cases, the XRD analysis revealed the formation of a hexagonal wurtzite ZnO structure with a predominant crystal plane of (002) on the surface of HDPE beads. Such crystal orientation is quite common using magnetron sputtering due to minimum surface energy. Nickel peaks were not detected by XRD. The surface chemical bond analysis of the ZnO film deposited on the HDPE beads without and with Ni underlayer confirmed the formation of Zn–O bonds in the wurtzite structure. Any additional elements except carbon, oxygen, and zinc were not detected by XPS. Although the morphology measurement showed a bit rough surface, any significant defects or detachments of ZnO film were not detected. Additionally, elemental mapping presented evenly distributed Zn and O across the surface of HDPE beads either without Ni or with Ni underlayer. Ni was observed only in the areas where ZnO film is possibly thinner or mechanically cracked.

Moreover, after testing under visible-light irradiation, it was verified that ZnO-coated HDPE beads with Ni underlayer (compared with without Ni) had an improved performance in the inactivation of bacteria alone and their mixture. The viability of *M. Luteus* and *S. Typhimurium* was reduced by 32% and 53%, respectively, when the bacteria were tested alone using photocatalyst with Ni underlayer. Meanwhile, the nickel-free photocatalyst decreased the viability by 25% and 28%, respectively. The viability of bacteria in the mixture demonstrated even better results. Surprisingly, the best result was achieved using ZnO-coated HDPE beads without Ni underlayer which reduced the viability of *S. Typhimurium* in the mixture with *M. Luteus* by 89%. Such increased bactericidal effect could be measured due to the negative interaction of different types of bacteria on each other. Investigation of infectivity of separate *PRD1* and *T4* bacteriophages and their mixture did not show a considerable difference between the ZnO photocatalysts without Ni and with Ni underlayer. However, it has been observed that a *T4* phage undergoes a greater photocatalyst-induced inactivation.

## Data Availability

Data are contained within the article.
